# Digestion of protein and toxic gluten peptides in wheat bread, pasta and cereal and the effect of a supplemental enzyme mix

**DOI:** 10.3389/fnut.2022.986272

**Published:** 2022-09-08

**Authors:** Daniela Freitas, Laura G. Gómez-Mascaraque, André Brodkorb

**Affiliations:** Teagasc Food Research Centre, Fermoy, Ireland

**Keywords:** Non-celiac Wheat Sensitivity (NCWS), Non-celiac Gluten Sensitivity (NCGS), Celiac disease, dietary supplements, gliadin, INFOGEST, semi-dynamic digestion

## Abstract

There has been an increasing interest in the relationship between wheat digestibility and potential toxicity to the host. However, there is a lack of understanding about temporal profile of digestion of wheat proteins from different food matrices under physiologically relevant conditions. In this study, digestion of three wheat-based foods (bread, pasta and cereal) was conducted based on the INFOGEST semi-dynamic protocol in the absence and presence of a commercial supplemental enzyme preparation (a Glutalytic^®^ based supplement, which will be marketed as Elevase^®^). Protein hydrolysis (OPA- ortho-phthalaldehyde - assay), molecular weight distribution (SEC-HPLC) and potential toxicity (R5 antibody-based competitive ELISA), were assessed. Our results demonstrated that under normal conditions, the complexity of the food influenced the temporal profile of protein hydrolysis and gluten breakdown throughout simulated gastric and intestinal digestion. However, treatment with the enzyme supplement significantly and acutely increased protein hydrolysis and gluten degradation in the gastric stage, and this enhanced digestion was maintained into the intestinal environment. These findings highlight the limitations of temporal gastric proteolysis and gluten degradation under normal conditions to different food types. They also show that supplemental enzyme mixes can effectively accelerate the breakdown of protein and hydrolysis of toxic gliadin fractions from the early stages of gastric digestion, thereby reducing intestinal exposure and potentially limiting the sensitization of the host.

## Introduction

The unique composition of gluten proteins is responsible for the multiplicity of their roles in our diets. From a culinary point of view, it enables the formation of unique structural features in staples such as leavened bread, pastries and pasta (1, 2), and is one of the reasons why cereals such as wheat have been used for millennia. From a nutrition and public health perspective, gluten is a common source of protein, however, its composition can pose a number of dietary challenges. Gluten proteins have traditionally been divided into two main groups according to their solubility in alcohol-water solutions (e.g., 60% ethanol) as (soluble) gliadins and (insoluble) glutenins, as reviewed by Wieser (3). They are made up of a complex mixture of hundreds of protein components (monomers, oligomers and polymers) and have a unique composition, with very low proportions of a number of essential amino acids, but with high contents of others, namely glutamine and proline, which combined constitute 50% or more of the total peptide-bound amino acids in gluten proteins (4).

Dietary gluten is considered the environmental trigger of Celiac disease (CD) in genetically pre-disposed individuals. Additionally, its ingestion can also have a negative impact in individuals who do not have CD. This has been described by the term Non-Celiac Gluten Sensitivity (NCGS) and can be defined as “one or more of a variety of immunological, morphological, or symptomatic manifestations that are precipitated by the ingestion of gluten in individuals in whom CD has been excluded” (5). Because it is also possible that non-gluten components of wheat, rye and barley trigger similar symptoms, the term Non-Celiac Wheat Sensitivity (NCWS) is currently preferred (6). It has long been observed that complete hydrolysis of gluten prior to ingestion seemed to remove its toxicity (7) but the ingestion of specific fractions of gluten digests obtained after hydrolysis by pepsin and trypsin were still deleterious for gluten-sensitive patients (8). The high repetition of glutamine and proline was later discovered to be particularly relevant here. Indeed, it is the abundance of these amino acids in specific protein segments that makes them extremely resistant to gastro-intestinal digestion, leading to the persistence of immunogenic peptide fractions such as the well-known 33-mer peptide of the α-gliadin (9, 10). If complete hydrolysis of gluten proteins prior to ingestion removes its toxicity, it is also likely that gluten toxicity would be eliminated if digestion were efficient enough to degrade all peptide fractions that can trigger CD or NCWS.

Protein digestion is initiated in the stomach by pepsin, and completed in the small intestine by pancreatic proteases and peptidases located in the brush border membrane (11). However, it is important to note that proteolysis does not start immediately after a meal. In fact, there can be significant delays to its start during gastric digestion because, depending on the meal, postprandial gastric pH can rise to 4.5 and up to 7 (12–14); i.e., well above the optimum pH for pepsin, which starts being active below pH 5.5, but only exhibits maximum activity at pH 2 (15). After a meal, gastric acidification occurs steadily and it can take between 60 and 200 min to reach optimum pH levels for pepsin (12–14).

It is generally agreed that protein digestibility, can influence the risk of allergenicity (16). It is also known that a number of different factors such as food processing and structure can influence the digestion of gluten proteins. It has been reported, for example, that the baking process reduces the digestibility of these proteins (17). The same has been found for pasta, as its manufacturing process creates a characteristically compact structure that protects gluten from proteolysis (18). Protease supplementation has been put forward as a potential aid, particularly in the context of accidental gluten ingestion (due to cross-contamination of gluten-free foods for example) (19).

Considering the dynamic aspects of gastric digestion and the complex interactions between food structure and protein digestion, the importance of *in vitro* studies that mimic dynamic digestive conditions and that include different gluten-containing foods as opposed to gluten isolates is clear. Recently, new protocols have been developed to enable us to mimic key aspects of digestion in humans more closely, particularly the dynamics of gastric pH, enzyme flow and emptying (20). However, there has been limited application of such systems to the study of gluten digestion and the potential role of digestive supplements. Therefore, the aim of this work was to study the temporal profile of digestion of three wheat-based foods produced through distinct processes and with different structures (bread, pasta and breakfast cereal) and to evaluate the impact of a commercial supplemental enzyme mix.

## Materials and methods

### Materials

Bread was prepared with white wheat flour (9.5% w/w protein, Supervalu, Ireland), spring water (Ballygowan Still Natural Mineral Water, Britvic Ireland Limited, Ireland), yeast (Belbake dried yeast, Lidl Ireland GmbH, Ireland) and table salt. Bead-shaped pasta (pasta spheres of ~3.5 mm diameter before cooking and 5 mm after cooking, made from white wheat flour, commercially available, Milaneza, Cerealis, Portugal) and a high fiber (10% w/w) wholegrain breakfast cereal (Weetabix Original, Weetabix Limited, UK) were purchased from local supermarkets. The supplemental enzyme mix used in this study was a Glutalytic^®^ based supplement specified in Supplementary Table 1, which will be marketed as Elevase^®^. This product was supplied by Deerland Probiotics & Enzymes (USA).

In addition to common analytical grade chemicals, the following products were used. Human saliva pooled from 10 individuals (ref. 991-05-P-250 from Lee Biosolutions, USA), porcine pepsin (P-6887) and pancreatin (P1750, 4USP) from Sigma-Aldrich (Ireland) were used in the digestion experiments. Two kits, the Pierce™ BCA Protein Assay Kit (catalogue number 23225, Thermo Scientific, Ireland), and the RIDASCREEN^®^ Gliadin competitive ELISA kit (article R7021 by R-Biopharm, Germany) were used in sample analysis protocols. Lugol's iodine solution (CH22380, Scientific Laboratory Supplies Ltd, United Kingdom) and Light Green SF Yellowish stain (0394-25G, VWR, Ireland) were used to prepare samples for optical microscopy.

### Food preparation and characterization

#### Preparation

Bread was made with 400 g of flour, 290 g of spring water, 3.5 g of dried yeast and 5 g of table salt in an automatic bread maker (Panasonic SD-2511) (Program 1, medium size, light crust). Pasta (25 g) was boiled for 11 min in previously salted spring water at a ratio of 10/1/100 (g of pasta/g of salt/g of water). Immediately after cooking, the water was drained and the pasta was rinsed with 250 mL of spring water 3 times. The breakfast cereal was used as purchased.

#### Characterization

##### Water content

This was determined by calculating the weight difference of food samples after drying for 24 h at 110 °C (DRY-Line^®^ - DL 53, VWR, Ireland).

Protein. The protein contents of the flour used to make the bread, of the pasta and the cereal were determined in duplicate by the accredited Teagasc Technical Services Lab at the Teagasc Food Research Centre, Moorepark using the Kjeldahl method (21, 22). For simplification purposes, the same conversion factor between nitrogen and protein was used for all foods, i.e., 5.7 (23).

##### Cryogenic scanning electron microscopy

Cryo-SEM was conducted following a protocol adapted from Ong et al. (24). Small pieces of the samples, cut with a blade, were mounted on copper holders using Tissue-Tek (OCT Compound; Sakura Finetek) to fix them, and immersed in liquid nitrogen slush for 15 s using an Alto 2500 cryo sample preparation system (Gatan, UK). The frozen samples were immediately transferred to the cryo preparation chamber of the Alto 2500 system, previously equilibrated at−140°C, through its vacuum transfer device. Specimens were then fractured inside the cryo preparation chamber using a scalpel blade, and etched for 30 min at−95°C. After cooling the chamber back to−140°C, samples were sputter coated with a gold/palladium alloy at 10 mA for 120 s and finally transferred under vacuum into the microscope. SEM was then conducted on a Gemini field emission scanning electron microscope (ZEISS, Germany) at an accelerating voltage of 2 kV and a working distance of 3.5–5 mm. Two detectors, an in-lens detector and a secondary electron detector were used to acquire the images.

### Proteolytic activity and stability of digestive proteases and the supplemental enzyme mix

To enable the comparison of the proteolytic activity of pepsin to that of the supplement, both products were tested using the standardized pepsin activity assay recommended by INFOGEST (25). This assay is based on the spectrophotometric determination of the TCA-soluble products obtained after incubating hemoglobin (substrate) with the enzyme at 37°C and at pH 2. Each sample was tested at least 5 times. Subsequently, to compare the proteolytic activity of the supplement to that of pepsin within the range of pHs to which they can be exposed during digestion, another set of experiments was conducted repeating the same assay with 10 substrate solutions prepared at different pHs from 2.5 to 7. For each pH, the supplemental enzyme mix was analyzed in duplicate and pepsin was analyzed in triplicate. One unit, as measured by this assay, will produce a ΔA_280_ of 0.001 per minute at pH 2.0 and 37°C, measured as TCA-soluble products.

### *In vitro* digestions

Semi-dynamic digestions of each of the three foods, both in the absence and presence of the supplemental enzyme mix, were carried out in triplicate. The pH-stat system described by Mulet-Cabero et al. (20) was used with methodological adaptations based on data from human studies as described below.

#### Oral phase

The two main components of mastication, comminution and saliva incorporation, were separated to facilitate bolus formation *in vitro*.

##### Food comminution

Bread (crumb only) was processed in a kitchen food chopper (Bosch CNHR 9EV 600 W, Bosch, Germany). Pasta was left intact as the size of the cooked beads (diameters between 3 and 5 mm or surface areas 7–20 mm^2^) was already close to that of particles obtained after mastication of other types of pasta. For comparison purposes, for masticated spaghetti and tortiglioni, the surface areas of most particles have been reported to range between 12–20 mm^2^ and 7.5–12.5 mm^2^, respectively (26). The cereal was manually crumbled because its brittle structure made it unsuitable for pre-treatment in a food processor. Separate portions of bread (15.0 g) pasta (31.3 g) and cereal (7.9 g), were stored in air-tight containers at room temperature until saliva incorporation (maximum of 6 h for bread and cereal and 3 h for pasta).

##### Saliva incorporation

Immediately before digestion was initiated, one portion of comminuted food was mixed with saliva (previously heated to 37 °C in a water-bath) for 30 s. The volume-to-mass ratio of saliva to food was 0.2, 0.05 and 1.0 (mL of saliva/g of food) for bread, pasta and cereal, respectively. The ratios for bread and pasta were defined according to previous work with the same types of foods (26). As this information was not found for this brand of cereal, data from wheat based toasts with a comparable moisture content was used as reference (27).

#### Gastric phase

##### Preparation

Each gastric digestion was carried out in a jacketed glass vessel (ref. 6.1418.250, Metrohm, Ireland) at 37°C. An overhead stirrer (OHS 200 Digital, VELP^®^ Scientifica, Italy or CAT R 100 CT, Ingenieurbüro CAT M. Zipperer GmbH, Germany) fitted with a 3D-printed stirrer head,Mulet-Cabero, Egger (20) was used to mix the chyme. To enable the pH to be measured from the start of the gastric phase, it was necessary to ensure an adequate volume of digesta. Therefore, the vessel was prepared with Simulated Gastric Fluid electrolyte solution (eSGF) at the same volume as that of the bolus obtained at the end of the oral phase for that food. This ensured compliance with the recommended oral-to-gastric contents dilution ratio (1:1) while allowing an adequate immersion of the pH probe. This pre-meal eSGF was prepared as described in the literatureMulet-Cabero, Egger (20) but without pH adjustment (i.e., at neutral pH) so as to allow the reproduction of gastric acidification rates observed *in vivo*. This eSGF was also used to prepare the two other solutions required for gastric digestion, pepsin and HCl (0.25 M). In preparation of these solutions, the eSGF was diluted 2-fold to ensure that the concentration of all electrolytes remained constant throughout the experiment.

##### Gastric digestion

Immediately after preparation, each bolus was introduced in the glass vessel and the chyme was stirred for 30–45 s at 20 rpm before stirring was interrupted and a first sample was collected. Stirring was resumed and gastric digestion was initiated by starting the addition of pepsin and HCl. Each gastric phase lasted 150 min. A total volume of 8 mL of pepsin solution was added from a 12 mL syringe at a rate of 0.053 mL/min using a syringe pump (ref. NE-4000, New Era Pumping Systems Inc., USA). Its concentration was pre-determined according to the gastric volume at t_0_ and the expected final volume to achieve a pepsin activity of 2,000 U per mL of digesta. HCl was added by a titrator (Metrohm Titrando 842 or Titrando 902) controlled by the TIAMO^®^ software (instruments and software from Metrohm, Ireland). Using TIAMO, the flow rate was continuously monitored in conjunction with gastric pH and adjusted as needed to first maintain a steady acidification rate of the digesta from its native pH (at t_0_) to pH 2 in 100 min (12, 14) and then keep it constant at 2 for 50 min. In addition to the first sample collected at t_0_ (after the oral phase and immediately before the start of gastric digestion), five other samples were collected during the gastric phase at 25, 50, 75, 100, and 150 min.

##### Gastric emptying

Gastric emptying was performed in three stages in each of which one portion of gastric digesta was pipetted using a 25 mL serological pipette with an opening between 2.07 and 2.2 mm wide and transferred to an individual tube for intestinal digestion. To standardize all the experiments, gastric emptying points were fixed at 50, 100 and 150 min. On each of the first two emptying points, the equivalent to 1/3 of the initial gastric volume was collected. On the last emptying point, all remaining chyme was collected (including any bigger particles that would not pass through the opening of the pipette).

#### Intestinal phase

##### Preparation

The intestinal fluids were composed of Simulated Intestinal Fluid electrolyte solution (eSIF), NaOH (2 M), pancreatin, CaCl_2_ (H_2_O)_2_ (0.6 mmol L^−1^) and water. The volume of eSIF (1.25 × concentrated) corresponded to 80% of the intestinal chyme. The volume of NaOH was that required to neutralize the pH of gastric digesta (as estimated from preliminary experiments). The activity of trypsin in pancreatin was determined in advance using the trypsin activity assay (25) and the concentration of pancreatin was adjusted accordingly to obtain a trypsin activity of 100 U/mL in the intestinal chyme. The volume of water corresponded to the remaining amount required to make up 100% of the total volume. For each gastric emptying point, one individual tube was prepared in advance with eSIF, NaOH and water.

##### Intestinal digestion

Immediately after gastric emptying, gastric digesta was transferred to the corresponding intestinal digestion tube and mixed by inversion. CaCl_2_(H_2_O)_2_ was added, the pH was verified and, if needed, adjusted. Finally, pancreatin was added and the tube was placed in a rotator (Stuart rotator SB3, Stuart Equipment, Cole-Parmer, UK) inside an incubator (Binder BF056, BINDER GmbH, Germany) at 37°C to initiate intestinal digestion. A 1:1 ratio of gastric digesta to intestinal chyme was aimed for, but their masses were recorded to allow for any necessary volume corrections in the final calculations. Each intestinal phase lasted 120 min, and two samples were collected at 10 and 120 min.

#### Samples

In summary, a total of 12 samples were collected during each digestion: one between the oral and gastric phases (t_0_), five during the gastric phase (at 25, 50, 75, 100 and 150 min) and two from each of the three intestinal digestions (at 10 and 120 min). All samples were collected in duplicates (two 1.5-mL aliquots) and heated to 80°C for 5 min for enzyme inactivation (the results of preliminary enzymatic activity tests after this heat treatment are presented in Supplementary Table 2). After cooling in ice, samples were stored at−20°C until required for analysis.

### Analysis of digesta samples

Before analysis, samples were thawed overnight at 4°C. One of the duplicates was used for optical microscopy. The other duplicate was centrifuged (3,000 *g*, 10 min) and the supernatant was recovered for the other analyses.

#### Microstructure

Optical microscopy was used to compare structural features of protein and starch in digesta samples. Micrographs were obtained using the 10x/0.40 and 20x/0.75 objectives of an Olympus BX51 digital microscopy system (Olympus Corporation, Japan) equipped with a ProgRes CT3 digital camera head (Jenoptik, Jena, Germany). ProgRes CapturePro software (v 2.10.0.0) was used for image capturing. The sample preparation method was adapted from a previously described protocol (28). Aliquots of bread and pasta digestion samples were stained directly whereas those of the cereal digestion were diluted 2-fold prior to staining. Staining was initiated by adding 500 μL of sample to 100 μL of aqueous Light Green solution (5 g/L) to color the protein. After 30 min, 60 μL of Lugol's solution was added to stain starch. One drop of each sample was deposited on a microscope slide and covered with a cover glass, immediately before analysis.

#### Protein release from the food matrix

The total protein released from the food matrix into the supernatant throughout digestion was quantified using the Bicinchoninic acid (BCA) protein assay. A commercial kit (Pierce^TM^ BCA Protein Assay Kit) was used according to the manufacturer's instructions. Briefly, 25 μL aliquots of centrifuged supernatant were pipetted into a microplate where they were mixed with 200 μL of the BCA working reagent provided with the kit. The covered microplates were incubated for 30 min, at 37°C and after cooling to room temperature the absorbance was read at 562 nm (Synergy HT, BioTek Instruments, USA). Whenever needed, samples were diluted with MilliQ^®^ water and the analysis repeated. Protein concentrations were determined against a calibration curve established with nine bovine serum albumin standards included in each microplate analyzed.

#### Protein hydrolysis

Proteolysis was monitored by quantifying the free amino groups using the previously described ortho-phthalaldehyde (OPA) spectrophotometric assay (29). Briefly, the OPA reagent was prepared by dissolving 3.81 g of sodium tetraborate in 80 mL of water and heating to 70°C while stirring for 20 min. After cooling, dithiothreitol (0.088 g) and SDS (0.1 g) were added. Finally, this was mixed with 0.08 g of OPA previously solubilized in 4 mL of absolute ethanol. Samples (10 μL) were then incubated with the OPA reagent in a microplate protected from light, at room temperature for 15 min, before an absorbance measurement was carried out at 340 nm. The concentration of free amine groups was determined against a calibration curve drawn from the results obtained with 7 leucine standards analyzed in the same microplate.

#### Protein molecular mass distribution

The molecular mass of proteins in the digesta was estimated by size exclusion chromatography, high performance liquid chromatography (SEC-HPLC) according to O'Loughlin et al. (30). Sample supernatants were prepared by filtrating through syringe filters with a polyethersulfone (PES) membrane with 0.45 μm pore size (Captiva Premium Syringe Filters reference 5190-5095, Agilent Technologies, UK). Briefly, a TSK G2000_SWXL_ column (600 × 7.5 mm; Tosoh Bioscience GmbH, Germany) with a guard column was used, in connection with a Waters 2695 HPLC with UV/Visible detector controlled by the software EMPOWER^®^. An injection volume of 10 μL was eluted at 0.5 mL/min with buffer (30% v/v Acetonitrile, 0.1% v/v TFA, prepared in HPLC grade water) and monitored over 70 min at 210 nm. Estimations were made using a calibration curve based on the retention times of the following standards bovine serum albumin, carbonic anhydrase, β-lg, aprotinin, bacitracin, histidine-leucine, phenylalanine and glycine, with respective molecular weights of 67000, 29000, 18400, 6500, 1400, 268, 165 and 75 Da.

#### Resistance of gluten fragments to digestion

The Ridascreen^®^ gliadin competitive ELISA kit was used to investigate the resistance of gluten proteins to digestion. The assay uses the R5 monoclonal antibody that recognizes toxic sequences are relevant to CD, particularly the QQPFP, and was approved by the American Association of Cereal Chemists to quantify gluten in hydrolysed material (method 38-55.01) (31–33). The assay was conducted according to the manufacturer's instructions on gastric samples collected at each gastric emptying point (50, 100, and 150 min) and on the corresponding intestinal phases. All samples were analyzed in duplicate. The instructions recommended appropriate dilution of the initial samples in 60% ethanol (v/v) therefore, the concentration of protein recovered in the ethanol-based solutions was also determined using the BCA assay as described in the Section Protein release from the food matrix.

#### Calculations and statistical analysis

During the digestion experiments, a total of 216 samples have been collected. One sample has been lost prior to any analysis (t0 sample of a bread digestion), another sample has been lost prior to analysis through the methods described in 2.5.5 (t150, digestion of cereal + supplement). Therefore, the corresponding data-sets have been excluded from the statistical analysis. With the exception of the data corresponding to these samples, all data are presented as means ± SDs.

The cumulated protein release and degree of hydrolysis at each time point of the digestion experiments have been calculated and normalized as a percentage of the total protein in the tested food. The influence of the protein content of the enzyme solutions used in the digestion experiments (pepsin, pancreatin and supplemental enzyme mix) on the results obtained with the BCA and OPA assays was accounted for in the calculations. Their contribution to the spectrophotometric endpoints of these assays was estimated based on previously determined calibration curves and on their concentration in the digestion samples. All statistical analyses have been performed using the Rstatix package (34) in R (version 1.1.463) (35) with statistically significant effects accepted at the 95% level. The normality of the results obtained from the analysis of digesta samples (BCA and OPA assays, respectively described in Section Protein release from the food matrix and Protein hydrolysis, and also the BCA and competitive ELISA assays carried out on the ethanolic fractions as described in Section Resistance of gluten fragments to digestion) was assessed using the Shapiro-Wilk's test. The homogeneity of variances was assessed using Levene's test. The homogeneity of variances has been confirmed for all data sets (*P* > 0.05). Normally distributed data sets were analyzed using a two-way mixed ANOVA (Rstatix package) with “time” as within-subject factor and “treatment” (food only or food plus supplement) as between-subject factor. Significant effects were observed for “time,” “treatment” and their interaction. Single factor ANOVA was then employed to evaluate if the presence of the supplemental enzyme mix produced, for each of the measurements performed, a statistically significant difference at each time-point of the digestion curves. For data sets that did not conform to the data normality assumption, a non-parametric test (Kruskal-Wallis test) was employed. When statistically significant differences were detected, *post-hoc* analysis of normally and non-normally distributed data was conducted using paired *t*-tests or Dunn's test, respectively.

## Results

### Food characterization

**Figure 1:**
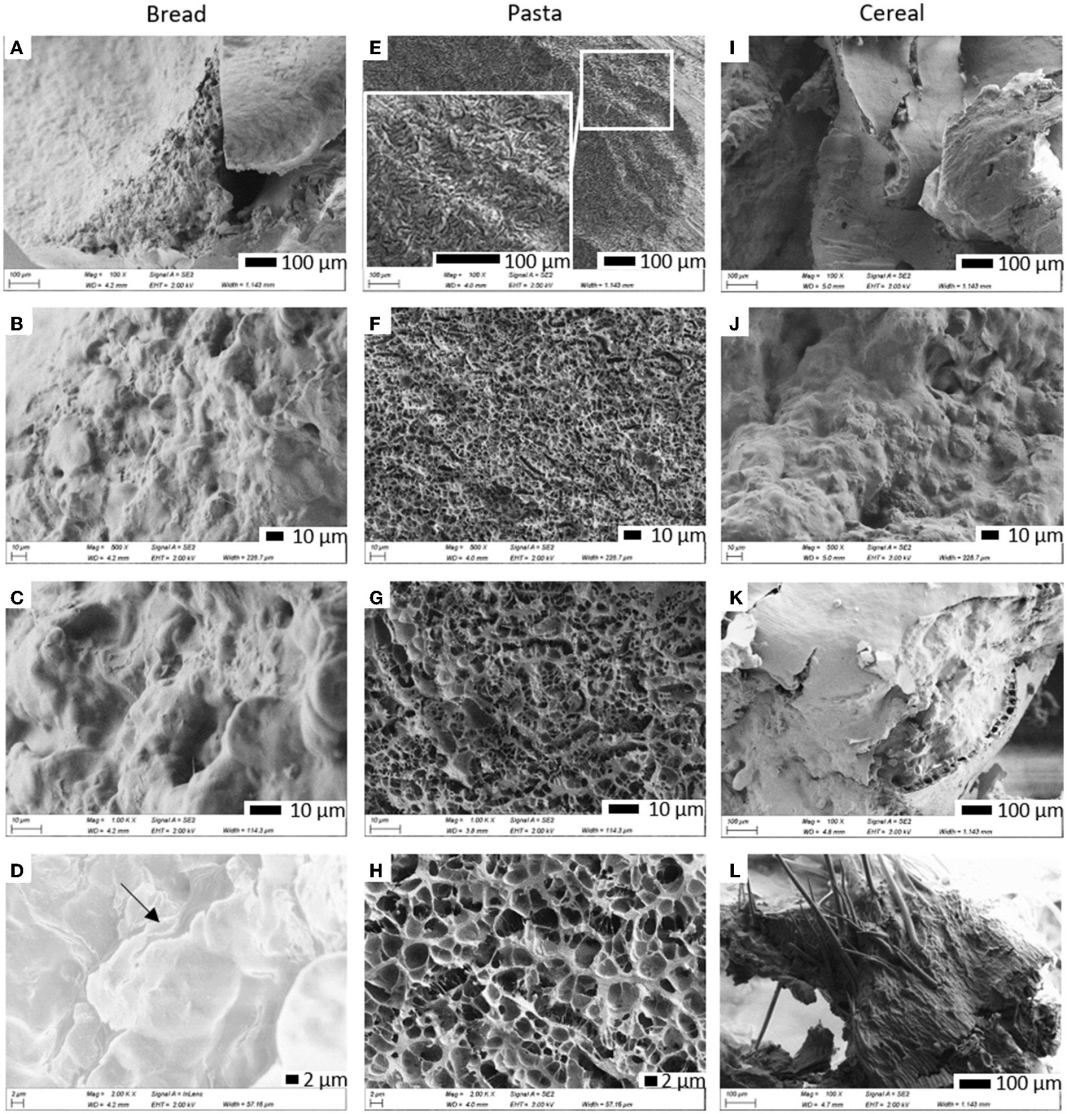
Scanning electron micrographs of bread, cooked pasta and breakfast cereal at different magnifications. Micrographs show cross-sections of bread crumb **(A–D)**, a cross section of pasta showing the border between its outer and internal surfaces **(E)** and only its internal structure **(F–H)** and a cross section of extruded wheat biscuit **(I–L)**. The arrow in **(D)** is pointing to starch leaching out of a starch granule.

The first set of experiments was aimed at characterizing the composition and microstructure of the foods tested. The composition of each food is presented in Table 1. As explained above, these foods were selected for having the same main ingredients but being processed differently, therefore having different structural features. SEM micrographs of the internal structure of these foods, obtained by sectioning and imaging the samples in cryogenic conditions, are presented in Figure 1. Images in the first, second and third columns correspond to cross-sections of bread crumb, cooked pasta and cereal, respectively. At higher magnifications, micrographs of bread crumb show a dense, continuous matrix in which embedded starch granules can be discerned (Figures 1B,C). Numerous starch granules retained their integrity and by using the in-lens detector it was possible to see gelatinised starch fractions leaching out of swollen granules (Figure 1D, black arrow is pointing to starch leaching out starch of a starch granule). The inner structure of pasta was characteristic of hydrogel networks, being composed of a porous system of interconnected filamentous material (Figures 1F,G) forming a honeycomb pattern visible at high magnifications (Figure 1H). The breakfast cereal greatly differed from both bread and pasta because it had a highly heterogeneous appearance. In some areas, a dense, continuous phase with embedded starch granules (Figure 1J) resembling that of bread (Figure 1B) could be observed. However, the microstructure of the cereal could be better described as an assembly of separate elements that retain native features of the wheat grain. This is in agreement with the higher fiber content of this food (Table 1), and can be illustrated by the wheat particles surrounded by the native aleurone layer (the outermost layer of the endosperm) (Figure 1K) or the persistence of the filaments that form the hairs of brush characteristic to the wheat grains (Figure 1L).

**Table 1:** Nutritional composition of bread, pasta and breakfast cereal.

	**Bread^a^**	**Cooked pasta^b^**	**Breakfast**
**(Flour)**	**(Raw pasta)**	**cereal**
Water content^c^ (g/100 g)	40.7 ± 0.2	74.7 ± 1.04	6.4 ± 0.1
	(10.7 ± 0.2)	(8.9 ± 0.1)	
Protein^d^ (g/100 g)	6.3 (9.5)	3.4 (12.4)	10.1
Carbohydrates^e^ (g/100 g)	50 (76)	19 (71)	69
Of which sugars	1.0 (1.5)	1.4 (5.0)	4.4
Dietary fibre^e^ (g/100 g)	2.0 (3.1)	0.8 (2.9)	10
Fats^e^ (g/100 g)	0.9 (1.3)	0.5 (1.8)	2.0
Of which saturated	0.1 (0.2)	0.1 (0.4)	0.6

^a^Except for the water content, the nutrient composition of bread was calculated from the composition of flour and the water content of bread and flour; ^b^Except for the water content, the nutrient composition of cooked pasta was calculated from the composition of dry pasta and the water content of both cooked and dry pastas; ^c^Oven-drying method; ^d^Kjeldahl method; ^e^Based on the product's label.

### Characterization of the supplemental enzyme mix

**Figure 2:**
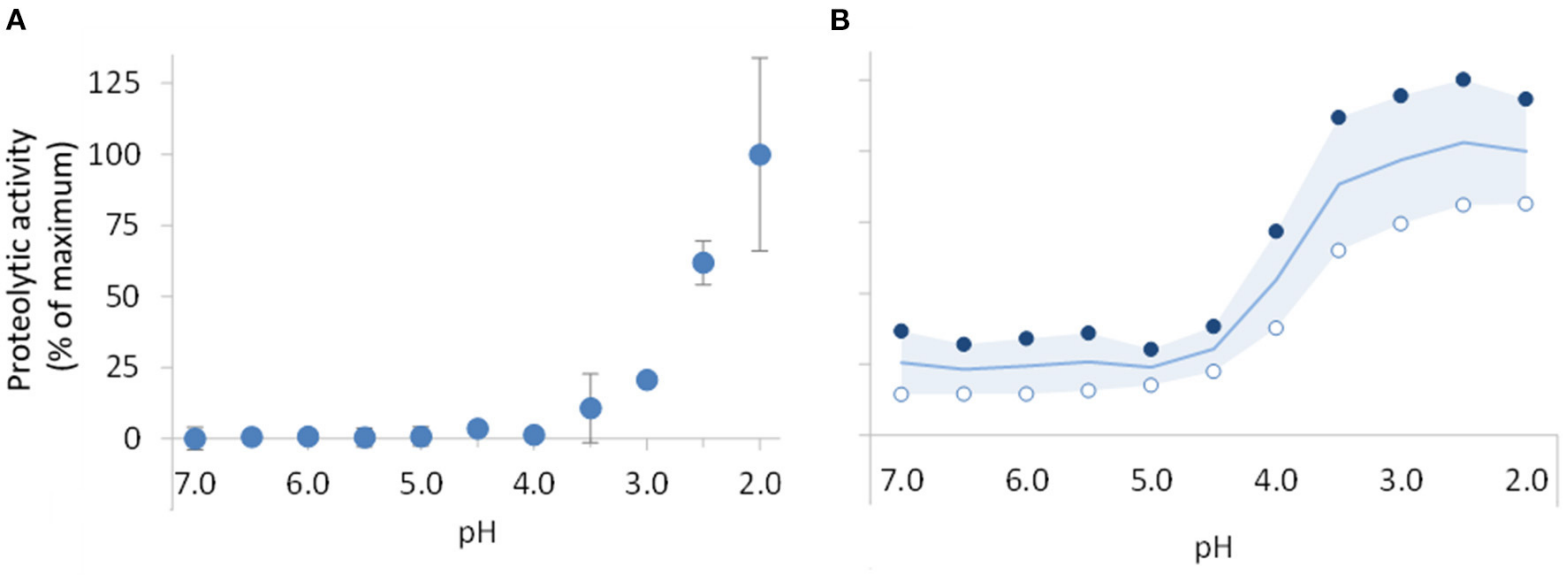
Impact of gastric pH reduction on the proteolytic activity of pepsin **(A)** and supplemental enzyme mix **(B)**. Proteolytic activity was determined using the pepsin activity assay recommended by INFOGEST (25) adapted to consider the different pH levels. Results are presented as percentage of the maximum activity, 3509 ± 687 and 240 ± 62 U/mg, respectively, for pepsin and supplemental enzyme mix, obtained with the standard protocol at pH 2. In chart **(A)**, data points are mean of 3 assays with pepsin ± SD. In chart **(B)**, open (°) and filled (•) circles correspond to series of results, respectively, obtained with the supplemental enzyme mix at concentrations of around 100 μg/mL and 1,000 μg/mL. The line and the colored area in this chart represent the mean of these data series and respective variance.

To obtain a comparative measure of the proteolytic action of the digestive enzymes and of the supplemental enzyme mix, the activities of pepsin and of the supplement were determined using the same assay, at 11 different pHs ranging from 7 to 2. This is representative of the acidity levels to which they can be exposed during gastro-intestinal digestion. The results are presented in Figures 2A,B for pepsin and the commercial supplement, respectively. As expected, pepsin was inactive at neutral pH and remained inactive when exposed to pH levels above 4. Below pH 4, proteolytic activity started being detected reaching 25% of the maximum activity at pH 3 and its maximum activity at pH 2. In contrast, the supplement exhibited proteolytic activity at all pH levels. Between pH 7 and 4.5, ~25% of the maximum activity was detected. Further pH reduction led to a sharp increase in proteolytic activity with maximum activity observed between pH 3 and pH 2. The maximum activities of the pepsin and supplement (standard protocol at pH 2) were 3,509 ± 687 and 240 ± 62 U/mg, respectively.

### Semi-dynamic digestions

#### Microstructural changes in digested samples

**Figure 3:**
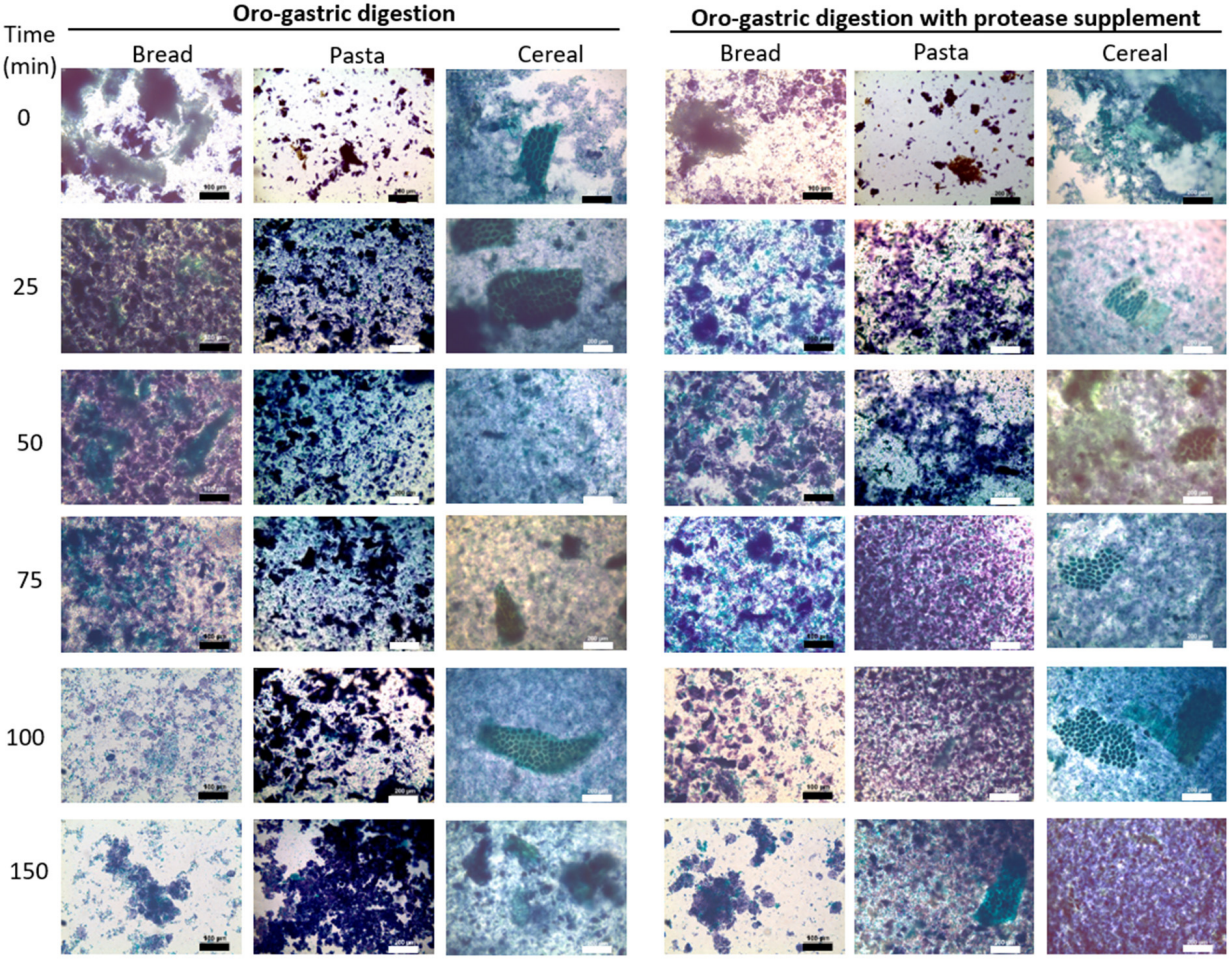
Light micrographs of samples collected at the end of the oral phase (t0) and throughout during gastric digestion (25–150 min) of bread, pasta and breakfast cereal. Micrographs on the right correspond to digestion experiments in which a commercial supplemental enzyme mix was added. Protein appears green and starch appears purple/blue due to staining with Light green and Lugol's solutions, respectively. For bread digestion samples, bar length represents 100 μm. For pasta and cereal digestion samples, bar length represents 200 μm.

Optical microscopy was used to monitor changes in structural features of the protein and starch fractions released from the food matrix during gastric digestion. The micrographs obtained are presented in Figure 3. Starch and protein appear purple/blue and green, respectively. With bread, a common feature in all the samples is the amorphous appearance. In the first 50 min, large protein agglomerates were observed, diffused among smaller starch fractions. Between 50 and 75 min there seemed to be a decrease in the size of the protein agglomerates which became evident after 100 min. At this point, only small protein agglomertes were observed and the remaining starch material often corresponded to seemingly intact starch granules. This contrasted with the digestion of pasta, for which the first 100 min of digestion were characterized by somewhat well-defined clusters of darkly-stained starch, surrounded by smaller starch and protein fractions. After 100 min, it became more difficult to differentiate protein (green) material and at the end of the gastric phase, similarly to what had been observed with bread, most material corresponded to starch granules. The samples of cereal digesta were more similar to those of bread in that they were also characterized by an amorphous appearance. Interestingly, the higher fiber content of the cereal enabled the identification of persistent differentiating structures. One example of a distinguishing feature of these samples, are the fragments of intact parenchyma native to the wheat grain. Such structures were visible in all samples of the gastric phase and also in intestinal digesta samples analyzed when the protocol was being set-up (Supplementary Figure 1).

With the addition of the supplemental enzyme mix there were visible changes in the digesta samples of bread and pasta which suggested a higher degree of degradation. During the digestion of bread, the protein fractions in the 25- and 50-min samples seemed to be smaller than those found in the digesta samples without the supplement collected at the same time-points. During the digestion of pasta, differences became evident after 75 min, when digested material appeared more amorphous, comparable to samples of bread digesta without the supplement at 75 min. For pasta, the structural differences seemed to persist until the end of the last intestinal digestion as it is suggested by pictures taken at this stage (Supplementary Figure 2) showing that for samples digested with the supplement there seemed to be less visible particles and those remaining appeared smaller. Optical microscopy did not seem to reveal any structural differences in the digestion samples of the cereal with the supplement.

#### Protein digestion

**Figure 4:**
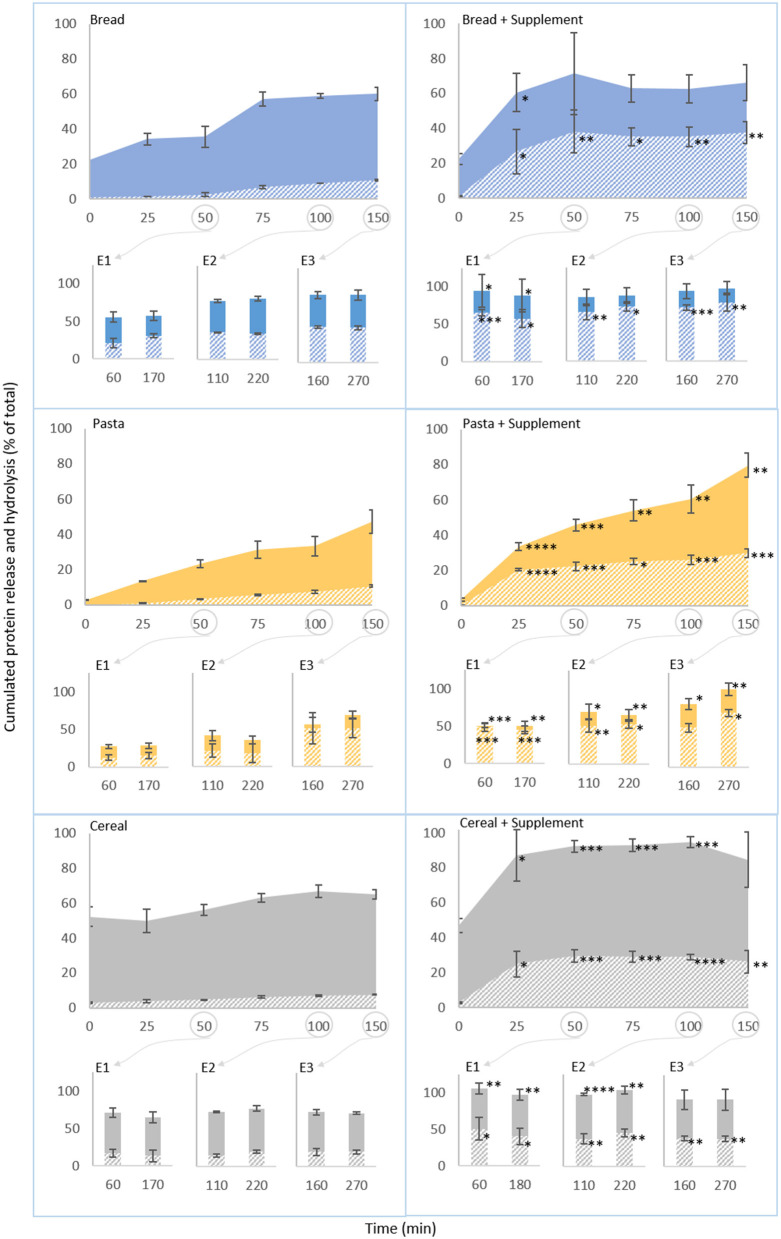
Semi-dynamic in vitro digestions of bread, pasta and breakfast cereal. Proportion of initial protein (%) released (filled areas) and hydrolysed (striped areas) during the course of digestions with equal portions of food. The first point in each curve corresponds to the end of the oral phase and the start of the gastric phase. The column charts present the results of intestinal digestion of gastric digested emptied at 50 (E1), 100 (E2) and 150 (E3) min. Results of the control experiments are presented on the left. Results obtained with the supplemental enzyme mix are presented on the right. Each data point represents mean ± SD, 3 rpt. The asterisk symbols denote significant differences compared to the digestion of the same food without the supplement (at the same time point): *P < 0.05, **P < 0.01, ***P < 0.001, ****P < 0.0001.

The BCA and OPA methods were used to determine the concentration of total protein and free amines in digesta samples, respectively, as measures of protein release from the food matrices and of protein hydrolysis. These results, plotted against time, are presented in Figure 4. Supplementary Table 3 summarizes the corresponding values, and Supplementary Figure 3 presents the gastric acidification kinetics and the consequent changes in proteolytic activity. There are six groups of charts in Figure 4. Within each group, data from oro-gastric digestions is presented in the larger chart on top, and gastric emptying times are indicated by circles linked to the corresponding intestinal phases. The filled and striped areas correspond to cumulated protein release and hydrolysis, represented as a percentage of the total protein in the food sample. The left column refers to normal digestions (without supplementation) of bread, pasta and cereal. As it can be observed, protein release patterns during oro-gastric digestion (filled areas, 0–150 min) varied with the type of food. At the end of the oral phase (t_0_) 22.6% ± 0.8% of the protein in bread had been released, this doubled during the gastric phase. First to 35.6% ± 6.1%, between t_0_ and 25 min (i.e., before any significant pepsin activation, according to Supplementary Figure 3B), and then between 50 and 75 min (when the gastric pH started enabling the activation of pepsin), from where it leveled off at around 60%. The concentration of free amines remained low and practically constant in the first 50 min of digestion (ranging between 5 and 7%), after which it increased steadily to reach 18.2 ± 0.3% by the end of the gastric phase. During the first 100 min of gastric digestion of pasta, protein release and hydrolysis was twice as slow as that of bread. Interestingly, protein release from the cereal matrix was about twice as fast as that of bread, however, the proportion of free amines remained about twice as low (comparable to that of pasta at the end of the gastric phase).

Intestinal digestion of all foods (bar-charts) led to an increase in protein release and hydrolysis in comparison to the corresponding gastric sample. For bread and pasta, protein release during intestinal digestion also tended to increase with each gastric emptying point, and the free amines followed the same trend. During the digestion of the cereal, however, the levels of protein released and free amines in the supernatant at all gastric emptying points were somewhat similar.

On the right-hand side of Figure 4, the results obtained with the supplemental enzyme mix are presented. As expected, the results obtained after the oral phase (t_0_, just before the supplement was added) were similar to those obtained without the supplement. However, after this, the supplement caused a marked increase on the proteolytic activity during the gastric phase (Supplementary Figure 3D). Consequently, significant increases of the proportions of protein released and hydrolysed from all foods have been observed throughout the gastric phase and at some intestinal phases too. These increases also occurred faster than any increase in the corresponding supplement-free experiment as reflected by the higher slopes of the curves in the charts on the right-hand side. The magnitude of these differences was particularly significant at the early stages of gastric digestions, where the levels of protein release and hydrolysis were comparable, and sometimes superior, to those found in the early stages of intestinal digestion (E1) of the same foods without the supplement. In the later stages of gastric digestion, protein release levels for bread were comparable to those found without the supplement, however, those of pasta and cereal were significantly higher (*P* < 0.01 and *P* < 0.001, respectively). By the end of the gastric phase, protein release represented 79.7 ± 6.7% and 84.6 ± 15.7% (vs. 47.1 ± 6.6% and 65.1 ± 2.8% without the supplement) of the total protein in pasta and cereal, respectively. The proportion of free amines was significantly higher at the end of gastric digestion of bread (*P* < 0.01), pasta (*P* < 0.001) and cereal (*P* < 0.01), what represented a 2- (bread) to 3-fold (pasta and cereal) increase compared to the digestions without the supplement. During intestinal digestions, there were also significant increases in the proportions of protein released and hydrolysed compared to the experiments without the supplement. As it can be observed, all the protein in bread and cereal was released from the food matrix after the first gastric emptying point (E1 after 50 min of gastric digestion, samples collected after 60 and 170 min of digestion), a significant increase (*P* < 0.05 and *P* < 0.01, respectively), from the results obtained in the analogous supplement-free experiments. The proportion of protein released from pasta at this stage was lower, but still about two times that observed without supplement (0.002 < *P* < 0.01), and it continued increasing with each gastric emptying point. Overall, in the last intestinal digestion (E3) the levels of protein released from bread and cereal in the presence of the supplement were comparable to those observed without the supplement, but remained significantly higher for pasta (0.004 < *P* < 0.05). At this stage, the levels of free amines were also significantly higher in most samples, reflecting a more extensive breakdown of wheat protein during supplemented digestions.

#### Molecular weight distributions of the proteins in the digesta

**Figure 5:**
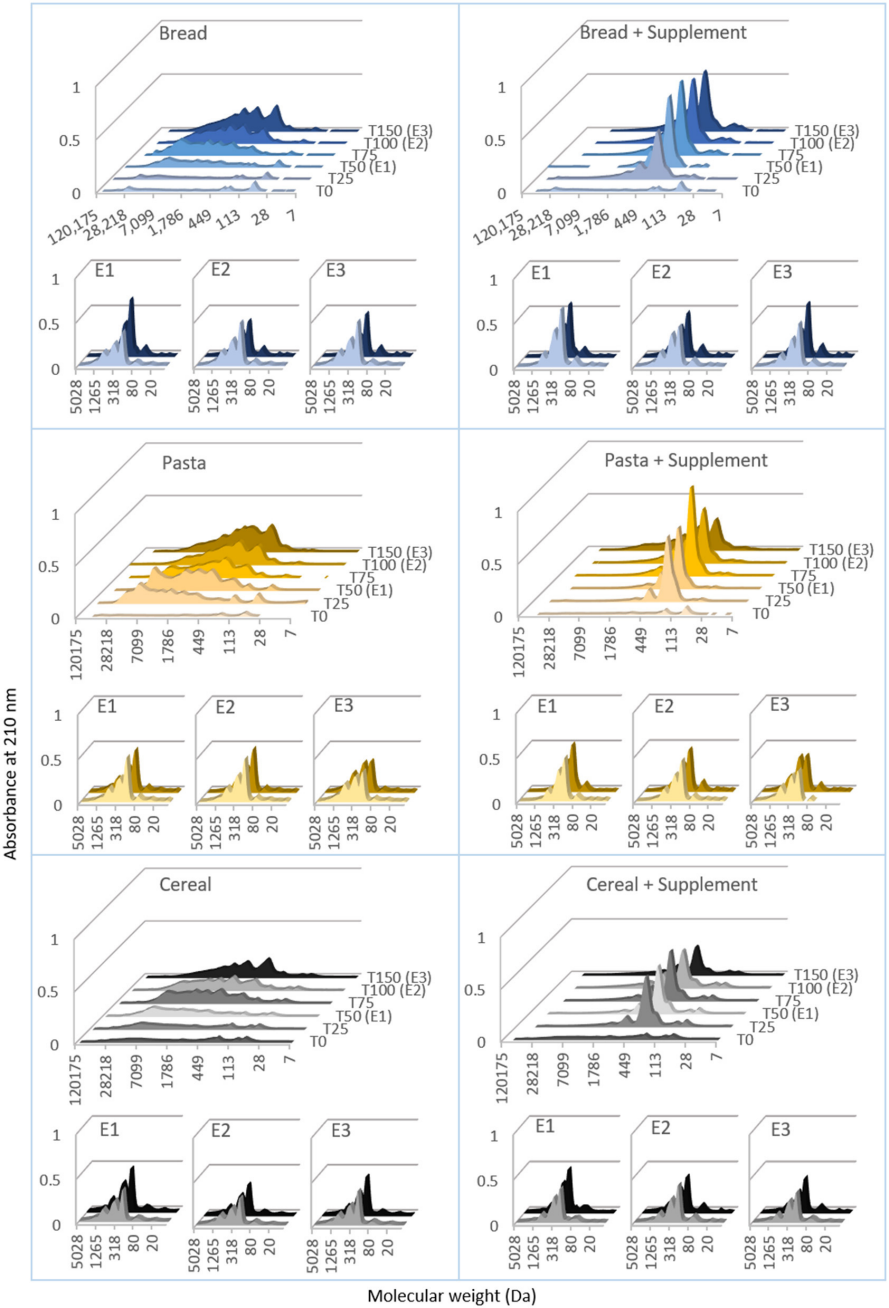
Molecular mass distributions of proteins during semi-dynamic *in vitro* digestions of bread, pasta and breakfast cereal. The molecular mass distribution of proteins in the digesta was estimated by size exclusion chromatography, high performance liquid chromatography (SEC-HPLC). The chromatograms show the absorbance at 210 nm, plotted against the molecular weight (estimated from the retention times observed with a set of standards). These results were obtained digesta samples collected during semi-dynamic digestions comprising gastric phase (t0 to t150) and three intestinal phases (E1, E2 and E3) conducted on gastric digesta emptied at three times points (50, 100 and 150 min). The left section presents results obtained with bread, pasta and breakfast cereal. The right section corresponds to digestion of the same in the presence of a commercial supplemental enzyme mix. Each curve was drawn from the averaged results of three samples.

SEC-HPLC was employed to estimate the molecular weight distributions of the proteins in the digesta samples. These results are presented in Figure 5, and the data is organized in the same way as in Figure 4. During gastric digestion of bread, the initial chromatograms were mostly flat, pointing to minimal peptide release. After 50 min of gastric digestion, the chromatograms started changing, presenting successively stronger signal intensities below 7,000 Da. Their shape remained fairly spread out until 150 min, when there was an increase in signal intensity in the region <2,000 Da, with a well-defined peak below 500 Da. During intestinal digestions, there was a shift in the chromatograms with all the signal concentrated below 1,200 Da and more well-defined peaks below 500 Da. The patterns found in the chromatograms obtained with the digesta samples of pasta and cereal were overall very similar to this. There seemed however to be a difference between pasta and bread, in that for pasta the chromatograms of gastric digesta started changing earlier than for bread (after 25 min of digestion, as opposed to 50 min). With the addition of the supplemental enzyme mix there was a clear change in the shape of the chromatograms of gastric samples. Taking bread as an example, it is possible to see that from the first 25 min of the gastric phase the chromatograms replicated the same pattern, being flat above 2,000 Da and with one sharp peak below 500 Da.

#### Potential toxicity of the proteins in the digesta

**Figure 6:**
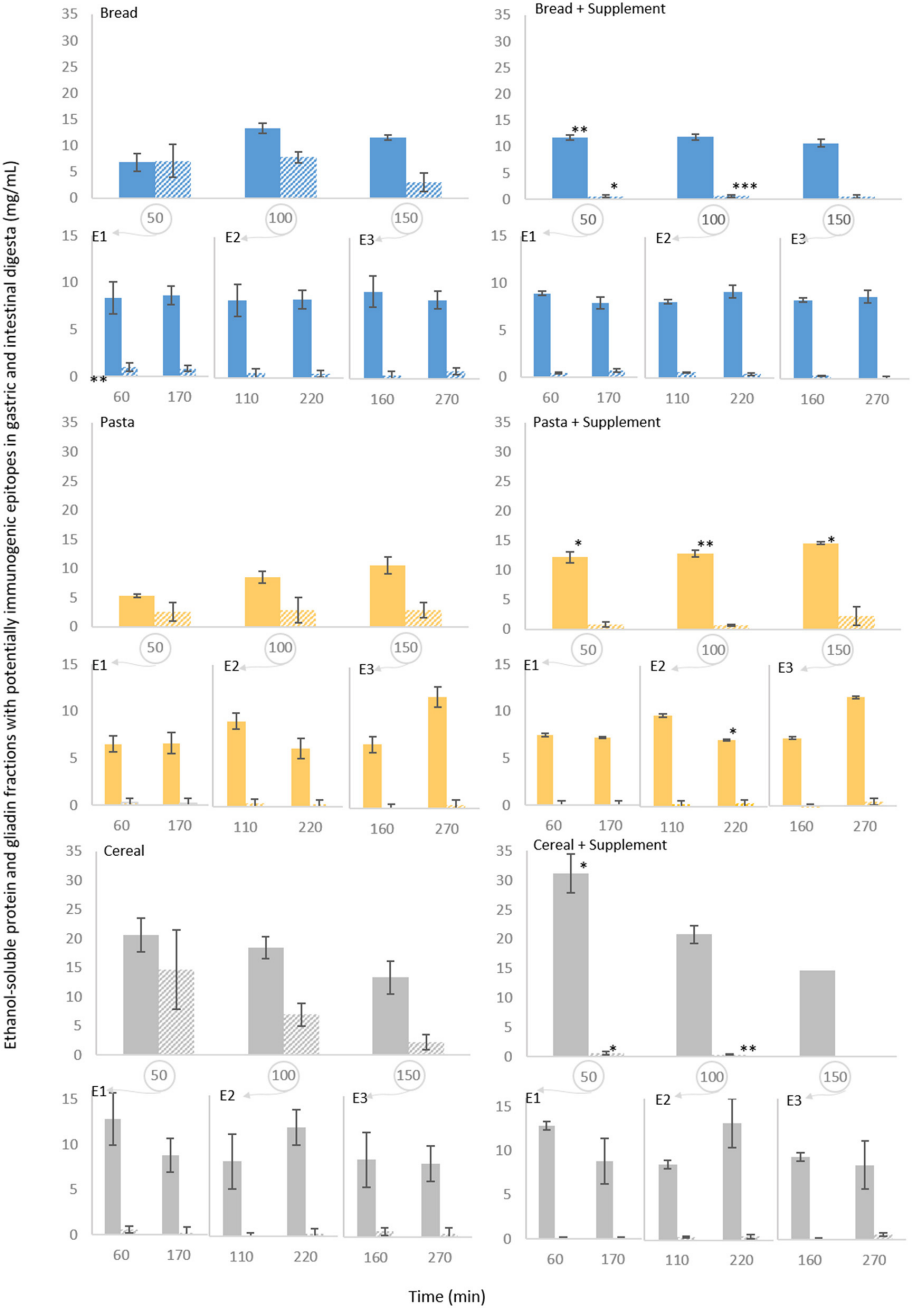
Persistence of specific epitopes during semi-dynamic *in vitro* digestions of bread, pasta and breakfast cereal. The results correspond to gastric digesta emptied at 50 (E1), 100 (E2) and 150 (E3) min and the corresponding intestinal digestions. The ethanol-soluble protein fraction (60% ethanol v/v) in the digesta samples was determined (filled columns) and its potentially toxic gliadin fractions were quantified (striped columns) by duplicate measurements using a competitive ELISA kit. Results of the control experiments are presented on the left. Results obtained with the supplemental enzyme mix are presented on the right. Each data point represents mean of three experiments ± SD, 3 rpt. The asterisk symbols denote significant differences compared to the digestion of the same food without the supplement (at the same time point): *P < 0.05, **P < 0.01, ***P < 0.001.

A competitive ELISA kit for hydrolysed gluten was used to quantify gliadin fractions with potentially toxic peptides recognized by the R5 antibody. To have a comparative measure between all samples, the protein soluble in the 60% ethanol (v/v) solution used for dilution of the digesta samples was also quantified. These results are presented in Figure 6. With bread, the concentration of ethanol-soluble protein ranged between 5 mg/mL at 50 min of gastric digestion (all of which quantified as gliadins) to between 10 and 15 mg/mL during the remaining gastric phase and subsequent intestinal digestions. Gliadin concentrations remained between 5 and 10 mg/mL until 100 min of gastric digestion, starting to decrease at 150 min, and reaching minimal to undetectable levels during intestinal digestion. Pasta digestion samples exhibited similar patterns. The highest concentration of ethanol-soluble protein and of gliadins was found in the samples of cereal digestion collected at 50 min (around 20 and 15 mg/mL, respectively). After 150 min, both the concentration of ethanol-soluble protein and of gliadin remained close to those found with the other foods.

While the concentrations of ethanol-soluble protein were significantly higher for the first 50 min of gastric digestion (with *P* < 0.01 for bread and *P* < 0.05 for both pasta and cereal) with the supplemental enzyme mix, there was a profound decrease in gliadin concentration to >1 mg/mL in all gastric and intestinal samples treated with the enzyme supplement. From the very first time point (50 min of gastric digestion) the concentration of gliadins with potentially toxic sequences was significantly lower for bread and cereal (*P* < 0.05), while a significant decrease in pasta was not observed likely due to variation and lower levels of gliadin in the pasta digestion supernatants.

## Discussion

### Impact of food characteristics on protein digestibility and potential health implications

The differences in the gastric digestive patterns of each food can be correlated to their distinct structural features. Overall, the highest levels of protein release were reached with the bread and cereal, however this occurred at different rates (Figure 4). For bread, the protein released into the supernatant during the gastric phase was about half of that of the cereal. This first release of protein could be attributed to the mechanical damage induced by the oral phase. The microscopic structure of bread (Figures 1A–D), characterized by a continuous phase made of a network of cross-linked gluten proteins and leached starch (primarily amylose) and a discontinuous phase of entrapped starch granules (2) has probably offered some resistance to further disruption of the matrix. For the cereal, the proportion of protein released into the supernatant after the oral phase (t_0_) was the highest of all three foods. The cereal matrix (Figures 1I–L) resembled a combination of individual components of the wheat grain which did not seem to form any type of network that could have conferred the type of resistance to mechanical disruption that was observed with bread. These structural features were also reflected in the product's brittleness and can be explained by the product's manufacture process, which is based on pressing together cooked and shredded wholegrain wheat. As digestion continued, there was only a slight increase in the protein released from the cereal but a more prominent release from bread immediately after the oral phase (between 0 and 25 min). This could be attributed to further disruptions of the food matrix, particularly starch-protein networks due to the ongoing hydrolysis of starch by salivary amylase because pH conditions at this stage did not yet support proteolytic activity. Pasta was not subjected to comminution as the size of beads was already representative of chewed particles of this type of food (26). This explains why its t_0_ samples presented the lowest proportion of protein in the supernatant. After this, similarly to bread, the gradual increase in protein release in the early stages of digestion (before pepsin activation) (Figure 4) can also be linked to the ongoing hydrolysis of starch in the outer and inner surfaces of the beads by salivary amylase. Indeed, its interconnected hydrogel network structure (Figures 1E–H) was similar to those observed in other types of pasta, namely spaghetti, and have been attributed to the formation of intricate associations between starch and protein (1).

The activation of pepsin (after 50 min, when the pH was reduced to below 4, Supplementary Figures 3A,B), enabled further protein release (particularly from bread and pasta) and the beginning of protein hydrolysis as indicated by the rise in free amines. It should be noted that despite more protein having been released from the matrices of bread and cereal than pasta throughout gastric digestion, protein hydrolysis levels remained fairly similar to those of pasta during the gastric phase. This suggests that the porous hydrogel structure of pasta may facilitate enzymatic access to protein despite it not being released from the food matrix. The experiments were designed to maintain a similar ratio of protein to proteases for all foods. Judging by the more extensive proteolysis with the protease supplement, a limited enzymatic activity and availability when only digestive enzymes were present cannot be ruled out. However, when additional digestive proteases were made available by the addition of pancreatin in intestinal digestions, the microstructural properties of the foods continued to influence the digestion of protein. What seems most interesting is the comparison of the endpoints of the intestinal digestions. Here, proportionally to the total protein in the food, pasta exhibited the closest relationship between the proportion of free amines and that of the protein released, followed by bread (which had an intermediate relationship, with roughly a 0.5 to 1 ratio) and the cereal. The limited proteolysis of bread proteins is in line with data showing that baking reduces the digestibility of gluten proteins within the bread crumb (17, 36). Indeed, it has been observed that heating wheat gluten can induce covalent protein aggregation resulting in a lower digestibility (37).

With the cereal, it was not only remarkable that there was a portion of protein that was never released after the oral phase, but also that the hydrolysis of the protein released was the least effective (as indicated by the lowest proportion of free amines). Two factors could have contributed to this (1). The native wheat structures in the cereal (Figures 1I,K,L) revealed to be highly resistant to digestion (Figure 3 and Supplementary Figure 1), probably having a protective effect and restricting protein release and hydrolysis (2). Additionally, the likely higher temperatures reached during the manufacture process of this food could have induced similar changes to those observed in the bread crust. The Maillard reactions and/or inter-peptide cross-linking that could have occurred here have been shown to impair even more the action of digestive proteases compared to the crumb (38).

A final remark could be made here regarding the value of semi-dynamic digestion protocols for these foods. If a static model had been used here, a long gastric digestion would have been carried out at a fixed, low pH and followed by one single intestinal phase. We could consider the final gastric emptying point (E3 at 150 min) and the samples from the subsequent intestinal phase (160 and 270 min) to be somewhat representative of a static protocol. It would be easy to conclude from it that there were only minor differences between each food in terms of exposure to potentially toxic sequences (Figure 6). However, under the more physiologically relevant conditions of the semi-dynamic approach, particularly the gradual decrease in pH, the overall exposure appears successively higher for pasta, bread and cereal, particularly due to differences in the first 50 min of gastric digestion that are linked with the structural properties of each food.

Gluten is present in numerous foods obtained through diverse manufacturing processes that result in distinct structural characteristics. Our work, suggests that this type of differences can influence digestive profiles and consequently also the way potentially toxic fractions are released from the food matrix and exposed during the passage in the gastrointestinal tract.

### Impact of supplementation

A number of *in vitro* (39, 40), animal (41) and human (42, 43) studies focusing on enzyme supplementation to boost the hydrolysis of gluten proteins have reported positive findings with this type of strategy. However, despite there being various commercially available products marketed as gluten digestive aids, it has been reported that several are ineffective in degrading toxic gluten fractions (44).

When analyzing the potential contribution of digestive supplements that are to be consumed with a meal, it is important to consider the impact of the changing gastric pH on their proteolytic capacity. Pepsin is inactive at the high pH levels found in the early stages of gastric digestion (Figure 2A) and depending on the buffering capacity of the food it can take 60 to 200 min for the pH to decrease enough to enable its activation (12–14). Meal supplementation with proteases that are active in these conditions will enable to at least anticipate proteins hydrolysis. Our results show that the supplemental enzyme mix tested is most effective below pH 4.5, however, it retains about 25% of its maximum proteolytic activity at higher pHs (Figure 2B). As such, when the supplement was added, there was a marked increase in the proteolytic activity at the early stages of digestion (Supplementary Figure 3D), which resulted in significantly higher proportions of protein being released and hydrolysed (Figure 4). Moreover, a significantly lower proportion of gliadin fragments recognized by the R5 antibody (Figure 6) during the early stages of gastric digestion was observed. Since gastric emptying can start as early as 10 min after a meal is initiated (45) the earlier start of proteolysis and more extensive degradation of potentially toxic gliadin fractions observed here can be helpful in reducing the exposure of the intestinal lumen to potentially sensitizing gliadin fragments.

## Conclusion

Our results highlighted the impact that food processing and subsequent structural changes can have on digestive processes *in vitro*. The structural differences between wheat-based bread, pasta and cereal had a marked impact in the digestion of protein at all stages of digestion. Overall, protein release from the food matrices was slowest for pasta and similar levels were reached with the bread and cereal. However, protein hydrolysis seemed to be most effective for pasta, which exhibited the closest relationship between the proportion of free amines and that of the protein released, followed by bread and the cereal. The impairment of proteolysis in bread was likely due to a reduction of protein digestibility related to structural changes during the baking process. In the cereal, the protection from proteolysis could have resulted from the combined effect of the formation of products highly resistant to digestion during the production process, and the preservation of native structures of the wheat grain in the final product which resisted all stages of digestion. Finally, this work has also shown how the absence of proteolytic activity at the early stages of gastric digestion can limit the breakdown of potentially toxic gliadin sequences. The supplemental enzyme mix tested was active from the beginning of the gastric phase and, consequently, it caused a significant increase in the proportion of protein hydrolysed from all foods, as well as a faster degradation of these toxic gliadin sequences. Given that gastric emptying commences prior to any protein hydrolysis under normal circumstances, the earlier start of proteolysis and more extensive gliadin degradation by the enzyme supplement could significantly reduce the exposure time and the concentration of sensitizers in the intestinal lumen, and potentially relieve unwanted intestinal discomfort.

## Data availability statement

The raw data supporting the conclusions of this article will be made available by the authors, without undue reservation.

## Author contributions

DF and AB contributed to conception and design of the study. DF performed experimental work, statistical analysis, and wrote the first version of the manuscript. LG-M performed experimental work. AB supervised the study. All authors contributed to manuscript revision, read, and approved the submitted version.

## Funding

This work was financially supported by Enterprise Ireland and Deerland Ireland R&D Ltd. (Part of Deerland Probiotics & Enzymes, USA) under an Innovation Partnership project (IP-2018-0711) awarded to AB. AB was funded by Science Foundation Ireland (SFI) and the Department of Agriculture, Food and Marine on behalf of the Government of Ireland under Grant Number (16/RC/3835). This research was supported using resources of the National Food Imaging Centre (NFIC) at Teagasc.

## Conflict of interest

Author DF was funded by IP-2018-0711. The findings of this research and manuscript have been shared with the funders prior to publication. The funders had no role in study design, data collection and analysis, or decision to publish. The remaining authors declare that the research was conducted in the absence of any commercial or financial relationships that could be construed as a potential conflict of interest.

## Publisher's note

All claims expressed in this article are solely those of the authors and do not necessarily represent those of their affiliated organizations, or those of the publisher, the editors and the reviewers. Any product that may be evaluated in this article, or claim that may be made by its manufacturer, is not guaranteed or endorsed by the publisher.

## Abbreviations

CD: Celiac disease
eSGF: Simulated Gastric Fluid electrolyte solution
eSIF: Simulated Intestinal Fluid electrolyte solution
NCGS: Non Celiac gluten sensitivity.
