# Supplementary material for: Digestion of protein and toxic gluten peptides in wheat bread, pasta and cereal and the effect of a supplemental enzyme mix
Source: Front Nutr. 2022 Sep 8;9:986272. doi: 10.3389/fnut.2022.986272 (PMC9493084; doi:10.3389/fnut.2022.986272)
Supplement: Supplementary file 1 [file Presentation_1.pdf]

Supplementary material

Table S1 - Composition of the supplemental enzyme mix. Components of the enzyme-based preparation including the enzymes and their sources, the total enzymatic activity of each enzyme in one tablet and the definition of the enzyme units used as provided by the manufacturer.

Component	Source	Units	Amount (mg)
Alpha Galactosidase	Aspergillus niger	150 GalU[1]	-
Amylase	Aspergillus oryzae	1,200 DU[2]	-
Beta glucanase	Trichoderma longibrachiatum	15 BGU[3]	
Cellulase	Trichoderma longibrachiatum	500 CU[4]	-
Diastase	Aspergillus oryzae	1,200 DP[5]	
Endo-peptidase Complex	Aspergillus niger, Bacillus sp.	75,000 HUT[6]/ 500 SAPU[7]	-
Exo-peptidase Complex	Aspergillus oryzae	125 DPPIV[8]	-
Glucoamylase	Aspergillus niger;	5 AGU[9]	-
Invertase	Saccharomyces cerevisiae	100 SU[10]	-
Lactase	Aspergillus oryzae	500 ALU[11]	-
Lipase	Candida rugosa	500 FIP[12]	-
Protease	Bacillus sp.	5,500 PC[13]	
Rice dextrin	-	-	79.8
Rice Bran	-	-	25.0
Xylanase	Trichoderma longibrachiatum	500 XU[14]	-

[1]GalU: α-Galactosidase Units - One unit is the quantity of the enzyme that will liberate p-nitrophenol at the rate of 1μmol/minute under the conditions of the assay (pH 5.5 and 37°C).
[2]DU: α-amylase dextrinizing unit - One unit is the quantity of α-amylase that will dextrinize soluble starch in the presence of an excess of β-amylase at the rate of 1 g/h at 30°C.
[3]BGU: β-Glucanase Units - One β-glucanase unit is defined as that quantity of enzyme that will liberate reducing sugar (as glucose equivalence) at a rate of 1μmol/minute under the conditions of the assay (pH 6.5 and 40°C).
[4]CU: Cellulase Units - One cellulase unit is defined as the amount of activity that will produce a relative fluidity change of 1 in 5 minutes in a defined carboxymethyl cellulose substrate under the conditions of the assay (pH 4.5 and 40°C).
[5]DP°: Diastase Units - One unit of diastase activity, expressed as degrees diastatic power, is defined as that amount of enzyme contained in 0.1mL of a 5% solution of the sample enzyme preparation that will produce sufficient reducing sugars to reduce 5mL of Fehling's solution when the sample is incubated with 100mL of the substrate for 1 hour at 20°C.
[6]HUT: Hemoglobin Unit Tyrosine base - One HUT unit of proteolytic activity is defined as that amount of enzyme that produces a hydrolysate whose absorbance at 275nm is the same as that of a solution containing 1.10μg/mL of tyrosine in 0.006N hydrochloric acid in 1 minute under the conditions of the assay (pH 4.7 and 40°C).
[7]SAPU: Spectrophotometric acid protease units - One spectrophotometric acid protease unit is that activity that will liberate 1μmol of tyrosine per minute under the conditions specified (pH 3.0 and 37°C).
[8]DPPIV: Dipeptidyl peptidase units: One unit will produce 1.0 μmole of p-nitroaniline from Gly-L-Pro p-nitroanilide per minute in 100 mM Tris-HCl under the conditions of the assay (pH 7.6 at 37 °C).
[9]AGU: Glucoamylase Units - One unit of glucoamylase activity (Amyloglucosidase) is defined as the amount of glucoamylase that will liberate 0.1μmol/minute of p-nitrophenol from the p-nitrophenyl-α-Dglucopyranoside (PNPG) solution under the conditions of the assay (pH 4.3 and 50°C).
[10]SU: Sumner Units - One unit is the quantity of enzyme which will convert 1mg of sucrose to glucose and fructose in 5 minutes under the conditions of the assay (pH 4.5 and 20°C).
[11]ALU: Lactase Units - One unit is defined as that quantity of enzyme that will liberate o-nitrophenol at a rate of 1μmol/minute under the conditions of the assay (pH 4.5 and 37°C).
[12]FIP: One unit of enzyme activity is defined as that quantity of a standard lipase preparation (Fungi Lipase-International FIP Standard) that liberates the equivalent of 1μmol of fatty acid per minute from the substrate emulsion under the described assay conditions (pH 7.00 and 37°C).
[13]PC: Bacterial Protease Units - One unit is defined as that quantity of enzyme that produces the equivalent of 1.5μg/mL of L-tyrosine per minute under the conditions of the assay (pH 7.0 and 37°C).
[14]XU: Xylanase units - One unit is defined as the amount of enzyme which liberates 1μmol of xylose per minute under the conditions of the assay (pH 5.3 and 50°C).

Enzymatic activity unit definitions are based on the Food Chemicals Codex. Detailed information about each assay can be found in this publication. (National Research Council 1996. Food Chemicals Codex: Fourth Edition).

Table S2 – Impact of heating on enzymatic activity. The recommended INFOGEST assays (Brodkorb, Egger, Alminger, Alvito, Assunção, Ballance, et al., 2019) were used to determine the amylase, pepsin, and trypsin activities of saliva, supplemental enzyme mix, pepsin and pancreatin as appropriate to their roles in digestion. After a sample processing cycle composed of 5-minute heating, freezing and thawing. Control data are presented as average of at least 3 assays ± SD. Results obtained after heating are presented as a percentage of the control value. Hyphens "-" denote samples where no activity has been detected.

Sample (units)	Amylase activity			Trypsin activity		Pepsin activity	
	Saliva	Supplemental enzyme mix	Pancreatin	Pancreatin	Supplemental enzyme mix	Pepsin [1]	Supplemental enzyme mix
**Control** (U/mg)	194 ± 51	2 ± 1	32 ± 9	6 ± 1	2 ± 1	2078 ± 372	2 ± 1
**75 ºC** (% of control)	-	-	-	101 ± 9		-	-
**80 ºC** (% of control)	-	-	-	101 ± 9	-	-	-
**85 ºC** (% of control)	-	-	-	92 ± 10	-	-	-

[1] A different pepsin lot was used in this experiment, hence the different activity value compared Figure 2 in the manuscript.

Table S3 – In vitro digestion of protein in bread, pasta and cereal and impact of a supplemental enzyme mix. Protein release and free amines (calculated from leucine equivalents) during semi-dynamic digestions based on the INFOGEST protocol. Results are presented as proportion of the total protein in the initial food sample and correspond to the average of 3 assays ± SD.

Sampling time (min)			Bread		Bread with enzyme supplement	
			Protein released (% of total)	Free amines (% of total protein)	Protein released (% of total)	Free amines (% of total protein)
Gastric phase		0	22.6 ± 0.8	4.9 ± 0.8	22.5 ± 3.2	1.4 ± 0.2
		25	34.3 ± 3.4	4.2 ± 0.2	60.5 ± 11.0	26.8 ± 12.7
		50	35.6 ± 6.1	6.8 ± 2.5	71.5 ± 23.3	38.3 ± 12.3
		75	57.1 ± 4.1	11.9 ± 0.5	63.1 ± 7.8	35.3 ± 5.0
		100	58.9 ± 1.3	15.2 ± 0.3	62.7 ± 8.1	35.2 ± 5.4
		150	60.1 ± 3.6	18.2 ± 0.3	66.3 ± 10.2	37.7 ± 6.2
Intestinal phase	E1	60	55.5 ± 6.6	38.3 ± 15.1	94.1 ± 21.8	65.2 ± 4.2
		170	57.4 ± 5.8	53.7 ± 5.3	88.1 ± 22.0	57.5 ± 11.8
	E2	110	77.6 ± 2.1	46.5 ± 0.8	84.7 ± 11.3	65.1 ± 10.4
		220	81.0 ± 3.0	42.8 ± 0.8	87.6 ± 10.5	72.5 ± 6.2
	E3	160	89.3 ± 4.4	52.4 ± 2.7	93.4 ± 9.8	71.6 ± 3.7
		270	89.2 ± 6.6	51.6 ± 7.3	97.1 ± 8.7	78.5 ± 11.9

Sampling time (min)			Pasta		Pasta with enzyme supplement	
			Protein released (% of total)	Free amines (% of total protein)	Protein released (% of total)	Free amines (% of total protein)
Gastric phase		0	2.7 ± 0.2	0.1 ± 0.0	3.3 ± 1.0	0.2 ± 0.2
		25	13.4 ± 0.1	1.0 ± 0.3	33.6 ± 2.2	20.3 ± 0.7
		50	23.6 ± 2.2	3.3 ± 0.2	45.8 ± 3.3	22.2 ± 2.5
		75	31.3 ± 4.9	5.7 ± 0.5	53.9 ± 5.9	24.9 ± 1.8
		100	33.3 ± 5.7	7.4 ± 0.9	60.3 ± 7.9	25.8 ± 2.6
		150	47.1 ± 6.6	10.8 ± 0.7	79.7 ± 6.7	29.7 ± 2.6
Intestinal phase	E1	60	27.2 ± 2.3	12.5 ± 0.1	50.2 ± 2.6	48.7 ± 5.8
		170	28.6 ± 3.6	15.1 ± 0.7	49.7 ± 6.4	44.5 ± 5.1
	E2	110	41.3 ± 6.9	21.0 ± 0.3	69.8 ± 9.9	51.4 ± 9.1
		220	35.5 ± 5.1	17.3 ± 2.2	65.7 ± 7.8	53.4 ± 5.7
	E3	160	56.2 ± 9.9	51.1 ± 15.3	83.8 ± 7.2	52.2 ± 5.6
		270	68.8 ± 5.5	51.6 ± 11.3	103.5 ± 8.1	71.9 ± 4.8

Sampling time (min)			Cereal		Cereal with enzyme supplement	
			Protein released (% of total)	Free amines (% of total protein)	Protein released (% of total)	Free amines (% of total protein)
Gastric phase		0	52.3 ± 5.6	2.8 ± 0.4	46.9 ± 4.2	2.7 ± 0.3
		25	50.0 ± 6.8	3.7 ± 0.8	86.9 ± 14.7	24.9 ± 7.3
		50	56.3 ± 3.1	4.8 ± 0.4	92.4 ± 3.3	29.4 ± 3.5
		75	63.2 ± 2.5	6.2 ± 0.6	92.8 ± 3.6	29.1 ± 3.0
		100	66.9 ± 3.6	6.7 ± 0.4	94.7 ± 3.2	29.0 ± 1.5
		150	65.1 ± 2.8	7.5 ± 0.3	84.6 ± 15.7	26.3 ± 6.5
Intestinal phase	E1	60	71.1 ± 6.0	16.9 ± 5.0	105.5 ± 7.2	50.5 ± 15.2
		170	64.8 ± 7.1	13.3 ± 7.9	97.2 ± 7.2	40.1 ± 11.6
	E2	110	73.1 ± 1.5	14.5 ± 2.0	98.2 ± 1.8	37.7 ± 6.9
		220	77.7 ± 3.5	20.0 ± 1.9	104.0 ± 5.4	45.9 ± 5.3
	E3	160	72.4 ± 3.8	19.4 ± 4.9	91.1 ± 13.3	38.1 ± 3.1
		270	71.2 ± 1.3	19.5 ± 2.5	91.5 ± 14.4	37.7 ± 3.8

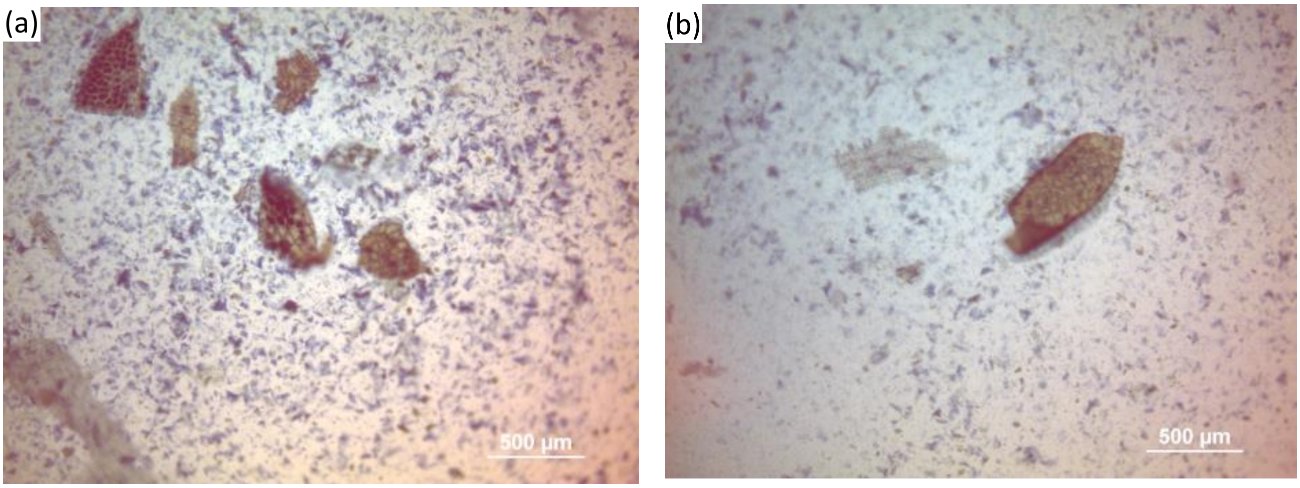

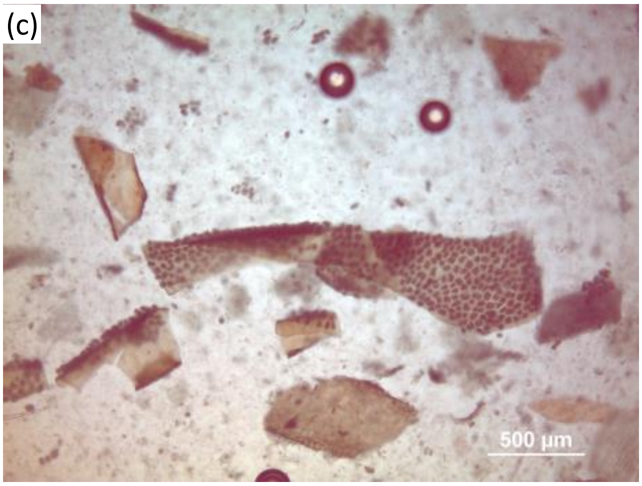

Figure S1 - Light micrographs of samples of digested wheat cereal in the last intestinal phase (after the last gastric emptying point). Micrographs on top correspond to samples of intestinal digestion collected at 160 and 270 minutes. The micrograph on the left corresponds to a sample collected at 160 minutes of a digestion experiments in which a commercial supplemental enzyme mix was added. Protein appears green and starch appears purple/blue due to staining with Light green and Lugol's solutions, respectively. Bar length represents 500 µm.

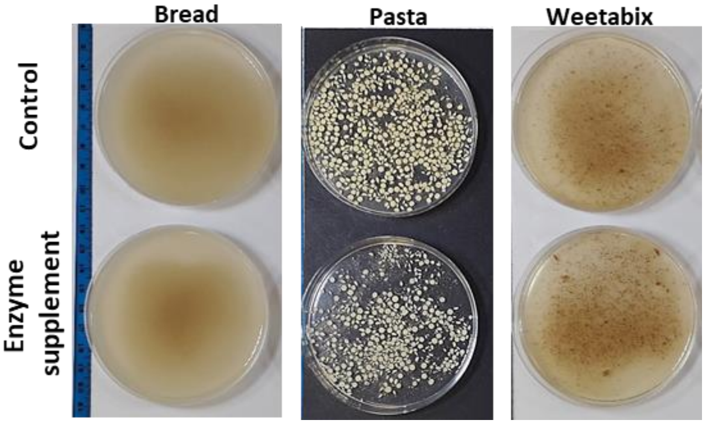

Figure S2 – Pictures of digested bread, pasta and weetabix. Samples obtained after complete *in vitro* digestion (oral, gastric and intestinal phases) of bread, pasta and weetabix in the absence and presence of a enzyme supplement.

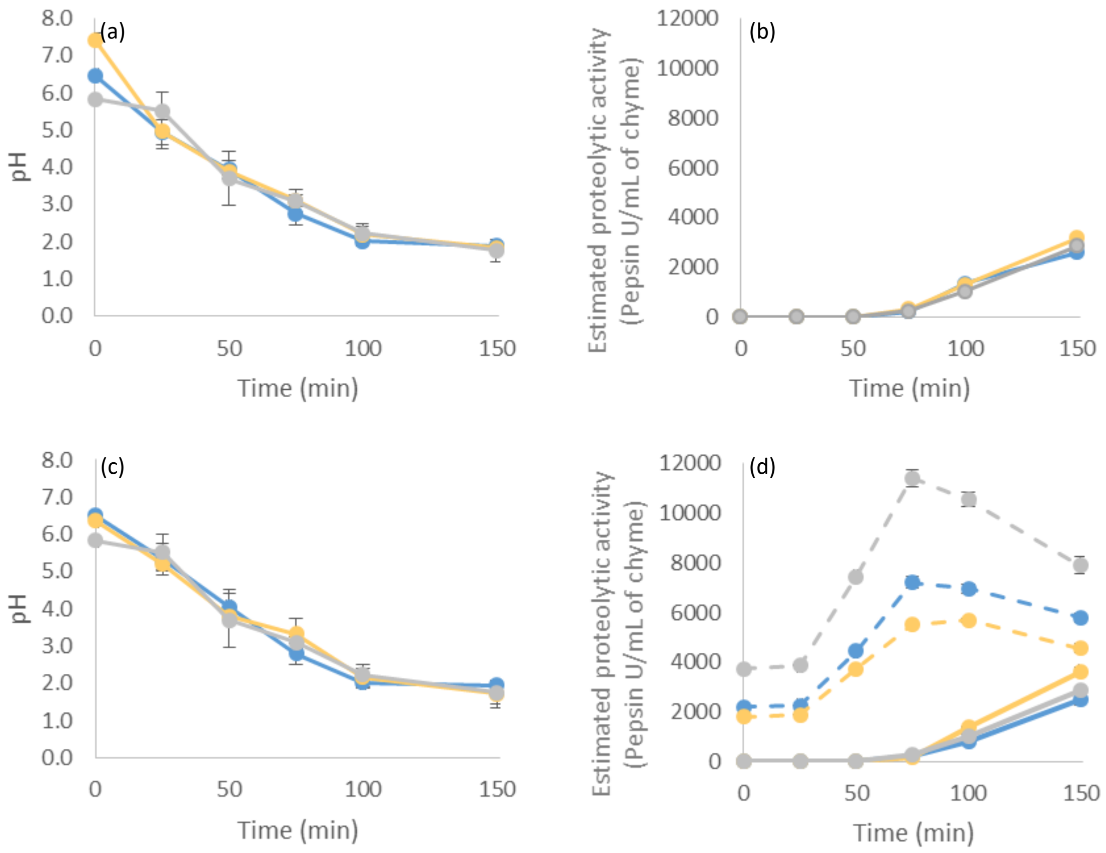

Figure S3 – Gastric pH and proteolytic activity during semi-dynamic digestions. Gastric pH at sample collection and gastric emptying time-points during semi-dynamic digestions conducted without (a) and with the enzyme supplement (c). The proteolytic activity in the digesta during the same experiments are presented in charts (b) and (d), respectively. These were estimated based on the pH curves in charts (a) and (c) and on previous results of pepsin activity assays (presented in Figure 2 in the article).
In charts (a) and (c), data points are mean of 3 digestions ± SD. In charts (c) and (b), filled lines correspond to pepsin and dashed lines correspond to the enzyme supplement. In all charts, the blue, yellow and grey curves correspond to results obtained with bread, pasta and cereal, respectively.
